# The Role of Biomarkers in Predicting Outcomes of Anterior Cruciate Ligament Reconstruction: A Systematic Review

**DOI:** 10.1177/23259671241275072

**Published:** 2024-10-07

**Authors:** Lachlan M. Batty, Christopher Mackenzie, Chelsea Landwehr, Kate E. Webster, Julian A. Feller

**Affiliations:** † OrthoSport Victoria Research Unit, Melbourne, Victoria, Australia; ‡ School of Allied Health, Human Services and Sport, La Trobe University, Melbourne, Victoria, Australia; § Western Health, Melbourne, Victoria, Australia; ‖ St. Vincent’s Hospital Melbourne, Melbourne, Victoria, Australia; ¶ Sunshine Coast University Hospital, Queensland Health, Birtinya, Queensland, Australia; Investigation performed at OrthoSport Victoria Research Unit, Melbourne, Victoria, Australia

**Keywords:** anterior cruciate ligament, biomarker, osteoarthritis, patient-reported outcome measures, blood, serum, urine, synovial fluid

## Abstract

**Background:**

Anterior cruciate ligament (ACL) injury is frequently associated with injuries to other parts of the knee, including the menisci and articular cartilage. After ACL injury and reconstruction, there may be progressive chondral degradation. Biomarkers in blood, urine, and synovial fluid can be measured after ACL injury and reconstruction and have been proposed as a means of measuring the associated cellular changes occurring in the knee.

**Purpose:**

To systematically review the literature regarding biomarkers in urine, serum, or synovial fluid that have been associated with an outcome measure after ACL reconstruction.

**Study Design:**

Systematic review; Level of evidence, 3.

**Methods:**

This review was performed according to the PRISMA (Preferred Reporting Items for Systematic Reviews and Meta-Analyses) guidelines. The MEDLINE, Embase, CINAHL, and Web of Science databases were searched to identify studies published before September 2023 that reported on patients undergoing ACL reconstruction where a biomarker was measured and related to an outcome variable. Of 9360 results, 16 studies comprising 492 patients were included. Findings were reported as descriptive summaries synthesizing the available literature.

**Results:**

A total of 45 unique biomarkers or biomarker ratios were investigated (12 serum, 3 urine, and 38 synovial fluid; 8 biomarkers were measured from >1 source). Nineteen different outcome measures were identified, including the International Knee Documentation Committee Subjective Knee Form, Knee injury and Osteoarthritis Outcome Score, numeric pain scores, radiological outcomes (magnetic resonance imaging and radiography), rates of arthrofibrosis and cyclops lesions, and gait biomechanics. Across the included studies, 17 biomarkers were found to have a statistically significant association (*P* < .05) with an outcome variable. Serum interleukin 6 (s-IL-6), serum and synovial fluid matrix metalloproteinase-3 (s-MMP-3 and sf-MMP-3), urinary and synovial fluid C-terminal telopeptide of type 2 collagen (u-CTX-II and sf-CTX-II), and serum collagen type 2 cleavage product (s-C2C) showed promise in predicting outcomes after ACL reconstruction, specifically regarding patient-reported outcome measures (s-IL-6 and u-CTX-II), gait biomechanical parameters (s-IL-6, sf-MMP-3, s-MMP-3, and s-C2C), pain (s-IL-6 and u-CTX-II), and radiological osteoarthritis (ratio of u-CTX-II to serum procollagen 2 C-propeptide).

**Conclusion:**

The results highlight several biomarkers that have been associated with clinically important postoperative outcome measures and may warrant further research to understand if they can provide meaningful information in the clinical environment.

Injury to the anterior cruciate ligament (ACL) results in many changes to the knee joint occurring at many different levels. In addition to changes to the biomechanical environment and joint kinematics, synovial fluid sampling studies suggest changes also occur at the cellular level very soon after ACL injury^22,27,30,43^ and ACL reconstruction.^22,30,35^

The measurement of biomarkers has been proposed as a means of evaluating these cellular changes, and there has been significant interest in the context of osteoarthritis outside of the ACL arena.^5,22,33,34,52^ In the osteoarthritis setting, it has been proposed that biomarkers may have a role in evaluating Burden of disease, be Investigative or Prognostic, have use in evaluating the Efficacy of an Intervention or a Diagnostic role - the BIPED approach as described by Bauer et al.^5^ Biomarkers with potential clinical utility have been broadly classified into biomarkers of collagen metabolism, biomarkers of aggrecan metabolism, biomarkers of noncollagenous proteins, and biomarkers of other processes such as inflammation.^42^

Posttraumatic arthritis after ACL injury is multifactorial in origin, but cellular-level changes are a part of this process.^9,20,39,44,67^ The concept of biomarkers predicting osteoarthritis is particularly attractive in this unique patient population who are typically young but at increased risk of developing osteoarthritis, and where disease onset and progression could potentially be identified and monitored before clinical or radiological signs become apparent. However, biomarkers are not only limited to evaluating osteoarthritis but may also have a role in predicting other outcomes after ACL reconstruction. Patient-reported outcome measures (PROMs),^7,29,36^ pain scores,^7^ and gait biomechanics^15,53^ are some examples of outcomes that have been associated with various biomarkers in the population who underwent ACL reconstruction.

Internationally, biomarker databases are being developed and tissue and fluid sample banks exist for patient cohorts with ACL injuries in Sweden,^58^ the Netherlands,^20^ the United States,^27^ and Australia,^2,16^ for example. Correlation of biomarkers to outcome variables over the long term is an emerging area and likely a future direction for research in patients with ACL injuries. The aim of this study was to systematically review the literature and synthesize the currently available evidence where biomarkers in blood, urine, or synovial fluid have been measured and associated with an outcome measure after ACL reconstruction.

## Methods

This review was performed according to the PRISMA (Preferred Reporting Items for Systematic Reviews and Meta-Analyses) guidelines and registered with the International Prospective Register of Systematic Reviews (PROSPERO; reference No. CRD42022343980).

### Search Strategy

A systematic search was conducted on March 18, 2022, and updated on September 4, 2023, in conjunction with a senior librarian at the institution at which the search was conducted. The following databases were used: MEDLINE, Embase, CINAHL, and Web of Science. Search terms were entered under 5 concepts: (1) anterior cruciate ligament, ACL, ACL reconstruction; (2) blood, plasma, serum; (3) synovial fluid; (4) urine; and (5) biomarker, biomarkers. In addition, 13 specific biomarkers of interest were searched for individually (eg, “CTX-2” and “interleukin”). The details of the search strategy utilized for MEDLINE are included in Appendix Table A1. To supplement electronic searches, the reference lists of relevant studies were also cross-checked for any additional references. The final list of candidate studies was then scanned and duplicates were removed. The results of the search were imported into Covidence.

### Selection Criteria

Studies were included in the review if they reported on associations between biomarkers from blood, urine, or synovial fluid and an outcome measure after ACL reconstruction. All reported outcome measures were included, grouped as “PROMs,”“radiological outcomes,” or “other.” We excluded reports on biomarkers after ACL injury without subsequent surgical management; animal studies; non–English-language studies; and reviews, commentaries, or conference proceedings/abstracts where no full text could be identified. Selection criteria were applied by 2 independent reviewers (C.M. and C.L.). A consensus was used to resolve any discrepancies, with a third reviewer (L.M.B.) adjudicating. All levels of evidence were considered.

### Methodological Assessment

The methodological quality of the included articles was assessed using the US National Heart, Lung, and Blood Institute (NHLBI) study quality assessment tools.^50,51^ The appropriate NHLBI tool was used for observational cohort studies and case-control studies.

### Data Extraction and Synthesis

Data were extracted concurrently by 2 reviewers (C.M. and C.L.) for the following variables: number of included patients and patient characteristics, details of the biomarkers measured including the time points of measurement, fluid sampled (urine, serum/blood, or synovial fluid), and biomarker levels. Details of the outcome measures were recorded including measurement/assessment protocols as well as the time points of the measurements.

Lotz et al^42^ provided a structure to conceptualize the numerous biomarkers that have been investigated in relation to osteoarthritis. This was published after a working meeting of the European Society for Clinical and Economic Aspects of Osteoporosis and Osteoarthritis. The authors consider 4 main biomarker groups: biomarkers of collagen metabolism, biomarkers related to aggrecan metabolism, biomarkers related to noncollagenous proteins, and biomarkers related to other processes (eg, inflammation and fibrosis). Where appropriate, the results in the present review are presented according to this structure to provide a framework for organization.

Given the heterogeneity of the included studies, no statistical analysis was performed. Data are presented in a descriptive manner and in tables where appropriate.

## Results

### Literature Identification

**Figure 1.**
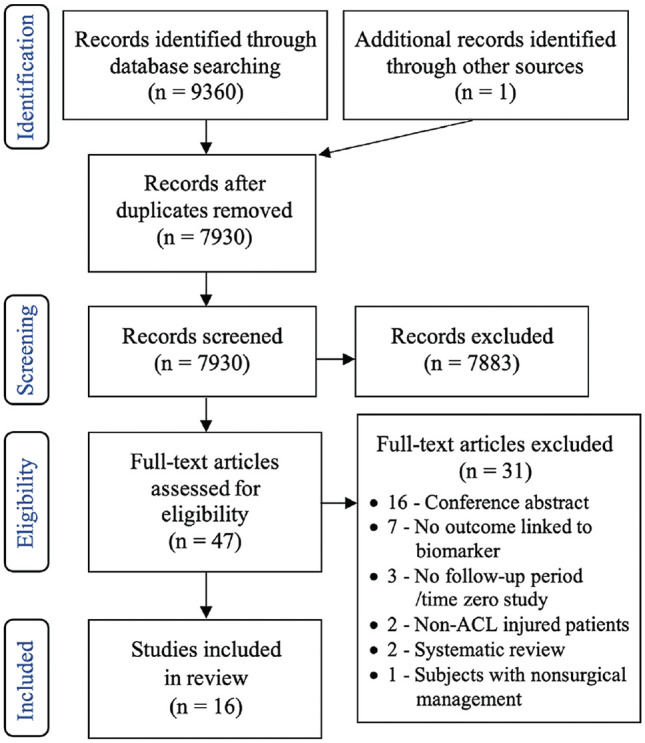
PRISMA (Preferred Reporting Items for Systematic Reviews and Meta-Analyses) flowchart of the study-inclusion process.

The electronic search yielded 9360 results, with a further single reference^59^ identified via citation tracking. After removing duplicates, 7930 titles and abstracts were screened, and 47 full-text articles were assessed for eligibility. A total of 31 results were excluded, some for multiple reasons, but the main reasons have been counted in this review (Figure 1). One paper combined operatively and nonoperatively managed ACL injuries and was excluded after we confirmed with the authors that no ACL reconstruction subgroup data were available.^59^

### Methodological Assessment

We identified 16 studies (492 patients who underwent ACL reconstruction) in which a biomarker was measured and linked to an outcome measure. There were 11 cohort studies,^¶^ 4 case-control studies,^4,7,15,32^ and 1 cross-sectional study.^54^ Ten studies had a prospective design,^#^ while 6 studies were retrospective.^4,25,32,44,54,56^ NHLBI quality-assessment scores ranged from 5^60^ to 9^56^ for cohort studies (maximum possible score, 14) and 7^7,32,63^ to 8^4,15^ for case series (maximum possible score, 12). Of the included studies, 0 were good, 12 were fair,^**^ and 4 were poor quality.^3,25,54,60^ Considerable heterogeneity of study design and outcome measures were identified, and it was decided that a meta-analysis was not appropriate.

### Overview of Included Studies and Description of Available Literature

Of the 492 included patients, the weighted mean patient age was 24.58 years (mean ages in the studies ranged from 18.4 to 34.04 years); 207 (42%) were female. Of the 16 studies, 9 studies (56%) took biomarker samples over multiple time points (33% took ≥3 samples), and 7 studies (44%) took samples at a single time point (70% of these were on the day of ACL reconstruction). A total of 45 unique biomarkers or biomarker ratios were investigated (42 biomarkers and 3 biomarker ratios). Of these markers, 12 were identified in serum, 3 in urine, and 38 in synovial fluid. Seven markers were measured in 2 different mediums, and 1 marker was measured in all 3 mediums. A summary of the biomarkers identified as well as a summary of their basic function is presented in Appendix Table A2; refer to Appendix Table A2 for expansions of abbreviations throughout the text. Any prefixes before the biomarkers indicate the source (s-, serum; sf-, synovial fluid; u-, urinary).

A summary of the identified biomarkers stratified by source (urine, synovial fluid, or serum) and functional grouping as per the Lotz et al^42^ classification is presented in Table 1. The majority of the biomarkers (n = 39) were measured in synovial fluid, with a wide variety of physiological processes including inflammatory pathways and metabolism of cartilage, bone, and synovium. In contrast, only 3 urinary biomarkers were identified, all being markers of chondral metabolism.

**Table 1:** Summary of Biomarkers and Biomarker Ratios Studied Across the 16 Included Studies^*a*^

	Biomarker Classification^*b*^					
Biomarker Source	Biomarker of Chondral Metabolism	Biomarker of Aggrecan Metabolism	Biomarker of Noncollagenous Proteins	Biomarkers of Other Processes (ie, Inflammation)		
Urine	**CTX-II** C1,2C C2C					
Serum	**C2C** C2C/CPII CPII	Aggrecan	**COMP** **MMP-3**	ALT AST	**IL-6** MCP-1 TNF-α **TTT**	
Synovial fluid	C2C C2C/KS CPII CTX-II NTX-1	C4S **C6S** **C6S/C4S** C2C/KS **KS** **sGAG**	BMP-2 COMP MMP-1 **MMP-3** **MMP-9** TIMP-1 TIMP-2	**bFGF** Bilirubin + biliverdin IFN-γ IL-1 **IL-1a** **IL-1b** **IL-1Ra**	IL-2 IL-4 **IL-6** IL-8 IL-10 IL-12p70 IL-13	**MCP-1** MIP-1B NO **RANTES** TNF-α TSG-6 **VEGF**

a Markers in bold have a statistically significant association with an outcome measure in at least 1 included study. See Appendix Table A2 for list of abbreviations.

b Per Lotz et al.^42^

### Outcome Measures

The outcome measures evaluated across the included studies are summarized in Table 2. There were 6 PROMs, 5 radiological outcome measures, and 8 outcome measures marked as “other,” including rates of arthrofibrosis and cyclops lesions, gait biomechanics and speed, ACL laxity measures, and arthroscopic assessment of chondral surfaces. Table 2 details the outcome measures used in the included studies and lists the biomarkers with and without an association demonstrated. Overall, the majority of biomarkers investigated were not found to be associated with the selected outcome variables (Table 2).

**Table 2:** Outcome Measures Used in the Included Studies^*a*^

Outcome	Biomarkers Investigated and Associated With Outcome During at Least 1 Time Point	Biomarkers Investigated and Not Associated With Outcome
Patient-Reported Outcome Measures		
VAS pain or NPRS	sf-IL-6, sf-CTX-II (Sullivan et al^60^) u-CTX-II (Chmielewski et al^7^) sf-IL-6, sf-IL-1 (Gupta et al^19^)	sf-IL-6, sf-IL-1Ra, sf-MIP-B, sf-MCP-1, sf-RATNES, sf-VEGF, sf-bFGF, sf-MMP-3, sf-TIMP-1, sf-TIMP2 (Markus et al^44^) sf IL-1β (Sullivan et al^60^) sf-TNF-α (Gupta et al^19^)
Lysholm score	sf-IL-6 (Gupta et al^19^)	sf-IL-6, sf-IL-1Ra, sf-MIP-B, sf-MCP-1, sf-RATNES, sf-VEGF, sf-bFGF, sf-MMP-3, sf-TIMP-1, sf-TIMP2 (Markus et al^44^) sf-IL-1, sf-TNF-α (Gupta et al^19^)
KOOS/KOOS-QOL	sf-IL-1α, sf-IL-1Ra, sf-MMP-9 (Lattermann et al^36^)	sf-IL-6, sf-IL-1Ra, sf-MIP-B, sf-MCP-1, sf-RATNES, sf-VEGF, sf-bFGF, sf-MMP-3, sf-TIMP-1, sf-TIMP2 (Markus et al^44^) sf-COMP, sf-CTX-II, u-CTX-II, sGAG, sf-IL-1β, sf-MMP-1, sf-MMP-3, sf-NTX-I, sf-TSG-6 (Lattermann et al^36^) s-MCP-1/s-COMP biochemical profile (Lisee et al^40^)
Tegner activity scale	sf-IL-6 (Gupta et al^19^)	sf-IL-6, sf-IL-1Ra, sf-MIP-B, sf-MCP-1, sf-RATNES, sf-VEGF, sf-bFGF, sf-MMP-3, sf-TIMP-1, sf-TIMP2 (Markus et al^44^) sf-IL-1, sf-TNF-α (Gupta et al^19^)
IKDC/IKDC-SKF	u-CTX-II (Chmielewski et al^7^) sf-IL-1α (Lattermann et al^36^)	sf-COMP, sf-CTX-II, u-CTX-II, sGAG, sf-IL-1β, sf-IL-1Ra, sf-MMP-1, sf-MMP-3, sf-MMP-9, sf-NTX-I, sf-TSG-6 (Lattermann et al^36^)
Marx activity scale	—	s-MCP-1/s-COMP biochemical profile (Lisee et al^40^)
Imaging-Based Outcome Measures		
Osteoarthritis (modified Outerbridge assessment per Colak et al^10^ on 3-T MRI)	sf-MCP-1, sf-VEGF, sf-IL-1Ra (Markus et al^44^)	sf-IL-6, sf-MIP-B, sf-RATNES, sf-bFGF, sf-MMP-3, sf-TIMP-1, sf-TIMP2 (Markus et al^44^)
Osteoarthritis (joint-space width on weightbearing radiograph per Dupuis et al^13^)	—	u-C2C/s-CP-II ratio (Tourville et al^63^)
Osteoarthritis (T1ρ and T2 quantitative assessment per Li et al^37,38^ on 3-T MRI)	High sf-GAG/low sf-IL-6; IL-8; IL-10; TNF-α; MMP-1; MMP-3 biochemical profile (Amano et al^3^)	High sf-IL-6; IL-8; IL-10; TNF-α; MMP-1; MMP-3/low sf-GAG biochemical profile (Amano et al^3^)
Osteoarthritis (MRI T1ρ relaxation times)	High s-MCP-1/s-COMP biochemical profile (Lisee et al^40^)	—
Tunnel enlargement (measured on plain radiograph)	sf-IL-6, sf-TNF-α, sf-NO (Zysk et al^69^)	sf-IL-1β, sf-BMP-2 (Zysk et al^69^)
Other Outcome Measures		
Arthrofibrosis (requiring manipulation or arthrolysis)	sf-RANTES, sf-bFGF (Avila et al^4^)	sf-IL-6, sf-VEGF-A, sf-TIMP-1, sf-IL-1Ra, sf-MMP-3, sf-MCP-1, sf-MIP-1B (Avila et al^4^)
Gait biomechanics (vertical ground-reaction force, knee flexion angle, internal knee extension moment)	sf-MMP-3, sf-IL-6 (Evans-Pickett et al^15^)	—
ACL laxity (KT-1000 arthrometer)	sf-IL-6 (Gupta et al^19^)	sf-IL-1, sf-TNF-α (Gupta et al^19^)
Physical Activity Score	sf-IL-1β (Inoue et al^25^)	sf-TNF-α, sf-IL-2, sf-IL-6, sf-IL-8, sf-IL-10, sf-IFN-γ (Inoue et al^25^)
Cyclops lesion	TTT (IgM) (Kodama et al^32^)	
Walking speed	s-C2C (Pietrosimone et al^54^)	s-Aggrecan (Pietrosimone et al^54^)
Gait biomechanics (peak vertical ground-reaction force, vertical ground-reaction loading rate, knee adduction moment)	s-IL-6, s-MMP-3, s-C2C/CPII (Pietrosimone et al^53^)	—
Osteoarthritis (arthroscopic chondral assessment)	sf-Δdi-C6S, sf-KS, sf-C6s/C4S ratio (Sobue et al^56^)	sf-C2C, sf-Δdi-C4S, sf-C2C/KS ratio (Sobue et al^56^)

a See Appendix Table A2 for a list of biomarker abbreviations. Biomarker prefixes: s-, serum; sf-, synovial fluid; u-, urinary. ACL, anterior cruciate ligament; IKDC, International Knee Documentation Committee; KOOS, Knee injury and Osteoarthritis Outcome Score; MRI, magnetic resonance imaging; NPRS, numeric pain rating scale; QOL, Quality of Life; SKF, Subjective Knee Evaluation Form; VAS, visual analog scale.

#### Patient-Reported Outcome Measures

PROMs assessed included the visual analog scale (VAS) for pain, Lysholm score, Knee injury and Osteoarthritis Outcome Score (KOOS), Tegner activity scale, International Knee Documentation Committee (IKDC) score, and Marx activity scale. Two studies found that higher baseline (time of surgery) synovial fluid interleukin levels correlated with worse Lysholm and Tegner scores at 12 months^19^ and lower levels of reaching the Patient Acceptable Symptom State for the IKDC and KOOS^36^ at 2 years. Increased sf-IL-6 at baseline was also associated with increased VAS pain scores in 2 studies with follow-up periods of 4 weeks^60^ and 1 year.^19^ Pain as measured by VAS had the highest number of biomarkers with a statistically significant association (Table 2). Lower u-CTX-II levels were associated with reduced pain levels in 1 study^7^; however, higher sf-CTX-II levels were associated with reduced pain in another study.^60^

#### Radiological Outcomes

Radiological outcomes of osteoarthritis were included in 4 studies (Table 2). Three were magnetic resonance imaging (MRI)–based assessments,^3,40,44^ and 1 was based on weightbearing radiographs.^63^ All 3 MRI studies used different outcome assessment protocols (Table 2). Of the 4 studies correlating biomarkers to radiological outcomes, the follow-up period ranged from 12 months^40^ to 7.8 years,^44^ and sample sizes were small, ranging from 18 patients^44^ to 35 patients.^63^ In the study with the longest follow-up (mean, 7.8 years), increasing levels of sf-MCP-1, sf-VEGF, and sf-IL-1Ra taken at the time of surgery were associated with increasing degenerative change on MRI at the final follow-up.^44^ In 1 study of 35 patients with ACL injuries, increased ratios of u-CTX-II/s-CPII (a ratio of type 2 collagen cleavage to synthesis) were associated with joint-space narrowing on weightbearing radiographs at the 4-year follow-up.^63^ One study of 24 patients demonstrated that increasing serum levels of MCP-1 and COMP (markers of inflammation and matrix degradation, respectively) between the preoperative and 6-month mark were associated with inferior cartilage composition on 12-month postoperative MRI.^40^

#### Other Outcome Measures

Three studies^15,53,54^ evaluating gait speed or biomechanics originated from the same center (Table 2). The authors found that patients with slower walking speeds had higher s-C2C concentrations.^54^ A stiffened knee gait strategy was associated with higher sf-IL-6 and sf-MMP-3 levels,^15^ and increased s-MMP-3 was associated with reduced limb symmetry indices in terms of knee adduction moment and peak vertical ground-reaction force loading rate.^53^

Two studies applying case-control methodology demonstrated an association between a biomarker and local or generalized arthrofibrosis, specifically a cyclops lesion formation^32^ or a postoperative procedure for stiffness.^4^ Increased synovial fluid biomarker RANTES as measured at the time of surgery was associated with increasing rates of manipulation under anesthesia/lysis of adhesions in 11 patients matched to 21 controls at a median of 92 days.^4^ High presurgery s-TTT values were associated with increased rates of cyclops lesion formation at 3 months postoperatively.^32^

One study investigated the association between biomarkers and knee stability as assessed by KT-1000 arthrometer testing.^19^ In a study of 59 patients who underwent ACL reconstruction, higher sf-IL-6 levels preoperatively were associated with poorer KT-1000 arthrometer laxity measures at 2, 6, and 12 months.^19^

### Biomarkers Summarized by Source (Urine, Serum, Synovial Fluid)

In terms of urinary biomarkers, there were 3 studies^7,36,63^ including 85 patients in which a urinary biomarker was measured. Three different urinary biomarkers were identified: u-C2C, u-C1,2C, and u-CTX-II; all are biomarkers of type 2 collagen degradation (Appendix Table A2). The mean follow-up across these 3 studies was 77.1 months (median, 46 months). Increased u-CTX-II correlated with increased pain scores over the short term (up to 16 weeks postsurgery) in a study of 28 patients who underwent ACL reconstruction.^7^ Increased u-CTX-II levels correlated with worse IKDC-SKF scores at up to a 16-week follow-up in the same study.^7^ Although not reaching statistical significance, increased u-CTX-II levels trended toward reduced rates of achieving the Patient Acceptable Symptom State for KOOS–Quality of Life scores (*P* = .08) at mean follow-up of 2.4 years in a series of 22 patients after ACL reconstruction.^36^

There were 5 studies^32,40,53,54,63^ with 113 patients overall (minimum sample size, n = 16^32^; maximum, n = 35^63^) in which a serum or blood biomarker was measured. Twelve different blood or serum biomarkers were reported on: s-aggrecan, s-ALT, s-AST, s-C2C, s-C2C:CPII, s-COMP, s-CRP, s-IL-6, s-MCP-1, s-MMP-3, s-CPII, and s-TTT. Where reported, the mean follow-up ranged from 6 months^53^ to 46 months.^63^ Increasing levels of s-MMP-3 and s-IL-6 were associated with reduced loading of the injured limb compared with the uninjured contralateral limb at a 6-month follow-up in a series of 18 patients using 3-dimensional gait analysis.^53^ Slower walking speeds were associated with higher s-C2C levels taken at 3-dimensional gait analysis assessment at a minimum of 6 months postoperatively in 20 patients.^54^ Increasing s-COMP and s-MCP-1 from the time of surgery to the 6-month follow-up was associated with inferior MRI-based cartilage proteoglycan density at the 12-month follow-up in a series of 24 patients.^40^

A total of 39 different synovial fluid biomarkers were identified in 10 studies^††^ reporting on a cumulative 351 patients (minimum sample size, n = 11^4^; maximum, n = 79^25^). The most frequently included biomarkers were sf-IL-6, sf-MMP-3, sf-IL-1b, sf-TNF-α, sf-IL-1Ra, and sf-CTX-II, which were reported in at least 3 separate studies. Where reported, the mean follow-up ranged from a minimum of 1 month^60^ to 7.8 years.^44^ Synovial fluid biomarkers of aggrecan metabolism, noncollagenous protein activity, and inflammation were associated with chondral degeneration and inferior PROMs in a number of studies. Baseline levels of sf-MCP-1, sf-VEGF, and sf-IL-1Ra correlated with increased MRI-assessed chondral degeneration at a mean follow-up of 7.8 years in 18 patients.^44^ Higher sf-sGAG concentrations (a marker of cartilage degeneration) at the time of surgery were associated with inferior cartilage composition on sequential MRI assessments during the first 3 years after ACL reconstruction in a study of 26 patients.^3^ Lower levels of sf-KS and sf-C6S/C4S at the time of surgery were associated with an increase in the number of high-grade cartilage lesions at 2-year arthroscopic assessment in a study of 62 patients.^56^

Overall, 17 biomarkers across 16 studies had a statistically significant association to an outcome measure on at least 1 time point. In contrast to Table 2, where the literature is summarized according to outcome measure, Table 3 highlights the 17 biomarkers found to have an association to an outcome measure and summarizes these findings. While Table 2 highlights the outcomes that can potentially be assessed with biomarkers in patients with ACL injuries, Table 3 highlights the specific candidate biomarkers that have shown some early promise.

**Table 3:** Biomarkers With a Statistically Significant Correlation or Between-Group Difference in Relation to an Outcome Measure in the Included Studies^*a*^

Biomarker	First Author (Year)	N	Follow-up	Key Findings
Biomarkers of Chondral Metabolism				
CTX-II	Chimielewski (2012)^7^	28	16 wk	u-CTX-II concentrations decreased over time and correlated with numeric pain rating scores at 4, 8, 12, and 16 wk postsurgery (*r* = 0.406; *P* = .039) Negative correlation between u-CTX-II concentrations and IKDC-SKF scores at 4, 8, 12, and 16 wk postsurgery (*r* = −0.402; *P* = .034)
	Sullivan (2023)^60^	23	4 wk	sf-CTX-II negatively correlated with VAS pain score over the first 4 wk postoperatively (*r* = −0.39; *P* = .002)
	Tourville (2013)^63^	35	4 y	Higher u-CTX-II/s-CPII ratios positively correlated with joint-space narrowing at 4 years on weightbearing radiograph; 11 patients with joint-space narrowing had significantly higher u-CTX-II/s-CPII ratios at 4 y compared with 31 ACL-intact controls
C2C	Pietrosimone (2016)^54^	20	43 ± 36 mo	s-C2C levels negatively correlated with walking speed (*r* = −0.52; *P* = .02), even after accounting for variance of stance phase duration (partial *r* = −0.53; *P* = .02)
	Pietrosimone (2017)^53^	18	6 mo	s-C2C/s-CPII ratios measured within 2 wk of surgery were negatively related to vertical ground-reaction force LSI at 6 mo postoperatively (*r* = −0.5; *P* = .04), but this was not significant after controlling for walking speed (*r* = −0.24; *P* = .36)
Biomarkers of Aggrecan Metabolism				
sGAG	Amano (2018)^3^	26	3 y	Patient group characterized by high sf-sGAG and low sf-IL-6, IL-8, IL-10, TNF-α, MMP-1, and MMP-3 positively correlated with higher T1ρ relaxation times (medial tibia: β = 3.29, *P* = .001; patella: β = 2.46, *P* = .007) and T2 relaxation times (medial tibia: β = 1.48, *P* = .32; patella: β = 1.74, *P* = .37) in the medial tibia and patella
C6S	Sobue (2017)^56^	62	2 y	Median baseline sf-Δdi-C6S levels at the time of surgery were significantly higher in the group who showed an increase in the number of high-grade cartilage lesions at 2-y follow-up compared with the group who did not show an increase in the number of high-grade cartilage lesions (53.4 vs 73.5; *P* = .004); high-grade cartilage lesions were defined as an increase in Outerbridge grade 3 or 4 lesions arthroscopically
KS	Sobue (2017)^56^	62	2 y	Lower sf-KS levels at the time of surgery were associated with an increase in the number of high-grade cartilage lesions at 2 years, as defined by increased Outerbridge grade 3 or 4 lesions arthroscopically (*P* = .021)
C6S/C4S	Sobue (2017)^56^	62	2 y	Lower sf-C6S/C4S levels at the time of surgery were associated with an increase in the number of high-grade cartilage lesions at 2 years, as defined by increased Outerbridge grade 3 or 4 lesions arthroscopically (*P* = .028)
Biomarkers of Noncollagenous Proteins				
Serum biochemical profile of increasing COMP and MCP-1	Lisee (2021)^40^	24	12 mo	Patients who had a serum biochemical profile of increasing s-COMP and increasing s-MCP-1 between the preoperative and 6-mo postoperative time points were associated with greater lateral femoral (β = 12.71; *P* = .04) and lateral tibial (β = 3.88; *P* = .001) MRI T1p relaxation times at 12 mo postoperatively; a *k*-means cluster analysis was used to create the different biomarker profile groups based on biomarker changes with time
MMP-3	Pietrosimone (2017)^53^	18	6 mo	Higher s-MMP-3 measured within the first 2 wk postsurgery correlated with reduced knee adduction moment LSI at 6-mo follow-up (*r* = −0.64; *P* = .01) Higher s-MMP-3 measured at 6 mo postoperatively correlated with reduced knee adduction moment LSI (*r* = −0.67; *P* = .01) and reduced vertical ground-reaction force loading rate LSI (*r* = −0.6; *P* = .01) at 6-mo follow-up
	Evans-Pickett (2021)^15^	38	6 mo	High sf-MMP-3 collected day 7 postinjury correlated with aberrant biomechanics at 6 mo postoperatively, including underloading and a stiffened knee gait strategy
MMP-9	Latterman (2018)^36^	22	2 y	Patients who failed to meet the Patient Acceptable Symptom state for the KOOS–Quality of Life had significantly higher sf-MMP-9 on the day of surgery compared with those did meet this (mean ± SD, 30.99 ± 35.96 vs 6.94 ± 10.30 ng/mL; *P* = .01; Cohen *d* = 1.07)
Biomarkers of Other Processes (ie, inflammation)				
IL-6	Pietrosimone (2017)^53^	18	6 mo	Higher s-IL-6 measured at the 6-mo postoperative mark correlated with reduced knee adduction moment LSI (*r* = −0.60; *P* = .02) after controlling for walking speed
	Evans-Pickett (2021)^15^	38	6 mo	High sf-IL-6 collected day 7 postinjury correlated with aberrant biomechanics at 6 mo postoperatively, including underloading and a stiffened knee gait strategy
	Gupta (2021)^19^	59	12 mo	Preoperative sf-IL-6 was associated with VAS scores, KT-1000 arthrometer testing, Lysholm knee scores, and Tegner scores at 12 mo; higher IL-6 preoperatively was associated with increased pain scores, decreased mechanical stability, and poorer Lysholm and Tegner scores
	Sullivan (2023)^60^	23	4 wk	sf-IL-6 was correlated with VAS pain scores preoperatively and over the first 4 wk postoperatively (*r* = 0.52; *P* < .001)
IL-1b	Inoue (2016)^25^	79	3 mo	Higher sf-IL-1b taken at 3-4 days postoperatively was seen in patients with a delayed recovery according to the authors’ 5-point ordinal recovery grading system (*P* = .03)
IL-1a	Latterman (2018)^36^	22	2 y	Patients who failed to meet the Patient Acceptable Symptom State for the KOOS–Quality of Life had significantly higher sf-IL-1a on the day of surgery (*P* = .004) Patients who failed to meet the Patient Acceptable Symptom State for the IKDC had significantly higher sf-IL-1a on the day of surgery (*P* = .02)
IL-1Ra	Latterman (2018)^36^	22	2 y	Patients who failed to meet the Patient Acceptable Symptom State for the KOOS–Quality of Life had significantly higher sf-IL-1Ra on the day of surgery (*P* = .03)
	Markus (2023)^44^	18	7.8 y	Lateral tibial plateau chondral lesion size as assessed by modified Outerbridge classification on 3-T MRI correlated with sf-IL-1Ra levels at the time of surgery (*R*^2^ = 0.271; *P* = .032)
MCP-1	Markus (2023)^44^	18	7.8 y	sf-MCP-1 levels at the time of surgery correlated with lateral femoral condyle chondral lesion depth (*R*^2^ = 0.362; *P* = .01) and size (*R*^2^ = 0.292; *P* = .025) as assessed by the modified Outerbridge classification on 3-T MRI
VEGF	Markus (2023)^44^	18	7.8 y	sf-VEGF levels at the time of surgery correlated with patellar chondral lesion depth (*R*^2^ = 0.606; *P* = .001) and size (*R*^2^ = 0.403; *P* = .006) as well as trochlear lesion size (*R*^2^ = 0.57; *P* = .001) as assessed by the modified Outerbridge classification on 3-T MRI
RANTES	Avila (2022)^4^	32	92 days^*b*^	sf-RANTES taken before surgical incision was significantly higher in the ACLR group with stiffness requiring manipulation under anesthesia or arthroscopic arthrolysis as compared with matched controls (OR, 2.28; *P* = .019)
bFGF	Avila (2022)^4^	32	92 days^*b*^	sf-bFGF taken before surgical incision was significantly higher in the ACLR group with stiffness requiring manipulation under anesthesia or arthroscopic arthrolysis as compared with matched controls (OR, 1.91; *P* = .047)
TTT	Kodama (2018)^32^	120	12 mo	Patients with cyclops nodule formation 12 mo after double-bundle hamstring ACLR had significantly higher TTT as measured at the time of surgery (OR, 9.34; 95% CI, 1.94-90.3; *P* = .002)

a Table stratified according to biomarker classification described by Lotz et al.^42^ See Appendix Table A2 for a list of biomarker abbreviations. Biomarker prefixes: s-, serum; sf-, synovial fluid; u-, urinary. ACLR, anterior cruciate ligament reconstruction; KOOS, Knee injury and Osteoarthritis Outcome Score; LSI, Limb Symmetry Index; MRI, magnetic resonance imaging; VAS, visual analog scale.

b Time to diagnosis of stiffness; actual follow-up time not stated.

## Discussion

This systematic review has demonstrated that multiple biomarkers in the blood, urine, and synovial fluid have been measured after ACL reconstruction and associated with clinically important outcome measures. However, this review suggests that there are not enough data on any of them to suggest that they are ready to be used in the clinical setting. The associations found are in the context of a plethora of biomarkers investigated across multiple series, typically with small patient numbers (median, 43; range, 13^69^ to 94^32^) and short follow-up periods (where reported: median, 9 months; range, 4 weeks^60^ to 7.8 years^44^). The chance of type 1 and 2 errors in the existing body of literature is high, and the methodological quality of the included studies was fair to poor. From a clinical perspective, the questions of what biomarker to measure, when to measure it, and what outcomes can reliably be predicted are far from having definitive answers.

Despite these challenges, this review highlights some encouraging results and a number of biomarkers worthy of further research. Serum IL-6, s-MMP-3, sf-MMP-3, u-CTX-II, sf-CTX-II, and s-C2C show promise in predicting outcomes after ACL reconstruction, Specifically, PROMs (s-IL-6,^19,60^ sf- and u-CTX-II^7,60^), gait biomechanical parameters (sf- and s-IL-6,^15,53^ sf- and s-MMP-3,^53^ s-C2C^54^), pain (s-IL-6,^19,60^ u-CTX-II^7,60^), and radiological osteoarthritis (u-CTX-II/s-CPII)^63^ were reported to be associated with these biomarkers.

In the existing literature, biomarker use has predominantly been explored as a prognostic tool. For example, biomarkers of chondral metabolism being used to predict future degenerative change ahead of time.^3,56^ Of note, a number of biomarker associations with specific short-term complications or outcomes such as cyclops lesion formation,^33^ arthrofibrosis,^4^ or postoperative pain^60^ were identified in this review. This may open the door for the concept of biomarkers being used as a management tool. Theoretically, serial preoperative measurements could guide surgical decision-making in terms of timing of surgery by waiting for biomarkers to reach a defined level. Preoperative sf-RANTES (associated with increased interventions for stiffness^4^) or s-TTT (associated with cyclops development^32^) concentrations could be monitored to optimize the timing of any surgical intervention. However, this concept assumes that levels normalize with time, something that is yet to be demonstrated. Although attractive, this would require serial biomarker measurements. Of the included studies, 56% (9/16) took samples over multiple time points, with 33% taking ≥3 samples. Only 38% (6/16) of the studies in this review took biomarker samples before ACL reconstruction, and no study assessed serial preoperative samples to define trends.

Patients undergoing ACL reconstruction represent a unique patient population. In this young and active population, there is estimated to be an 8-fold increase in the rate of osteoarthritis after ACL reconstruction, and the prevalence of osteoarthritis was estimated to be 36% at 10 years after ACL reconstruction in 1 umbrella systematic review.^66^ Predicting osteoarthritis with accuracy at the individual level is difficult. Rates of osteoarthritis vary widely, from 0% to 100% at 10 years in another systematic review,^39^ highlighting the inherent challenges of defining and investigating this phenomenon. The multifactorial origin of posttraumatic osteoarthritis makes determining the significance of individual risk factors difficult. Biomarkers represent an attractive way to try to better understand the pathophysiology of this process and would ideally predict who is at risk of developing premature posttraumatic osteoarthritis. This has been a focus of the research efforts to date, with 3 studies correlating biomarkers to chondral degeneration or osteoarthritis on MRI,^3,40,44^ 1 correlating with radiographic changes,^63^ and 1 with arthroscopic findings^56^ (Table 2). Furthermore, the IKDC, KOOS, and Lysholm PROMs were assessed across 5 studies^7,19,36,40,44^ (Table 2), which include an assessment of pain potentially reflecting degenerative change.

Six biomarkers or biomarker ratios (u-CTX-II/s-CPII, sf-MCP-1, sf-VEGF, sf-IL-1Ra, high sf-GAG/cytokine ratio, high s-MCP-1/COMP ratio) were associated with MRI-diagnosed chondral pathology.^3,40,44,63^ Although more data are needed, by aiming to directly measure chondral metabolism as a mechanism of osteoarthritis pathogenesis, biomarkers may reflect the influence of many, if not all, of the multiple factors contributing to the development of osteoarthritis. For example, in the multifactorial osteoarthritis development process, it is difficult to quantify the individual contributions of factors such meniscal injuries, residual instability, malalignment, or genetic factors, which are inherently heterogeneous and difficult to quantify. However, these factors may all contribute to a common pathway of chondral degradation that can potentially be measured directly with biomarkers of chondral breakdown. U-CTX-II, a breakdown product of type 2 collagen, appears to be a leading candidate for further investigation given the ease of collection from the urine and the association with radiological osteoarthritis,^63^ pain^7,60^ and PROMs^7^ demonstrated in the existing literature.

A 2015 systematic review by Harkey et al^22^ analyzed the available literature until June 2014 regarding osteoarthritis-related biomarkers after ACL injury and reconstruction. The authors identified 8 studies in which biomarkers of osteoarthritis were studied in the context of ACL reconstruction. The authors identified decreased levels of biomarkers indicating collagen breakdown in the serum, but increased levels of biomarkers indicating collagen breakdown in the urine after ACL reconstruction as compared with controls. When compared with preoperative values, synovial inflammatory cytokine biomarkers increased, while plasma biomarkers did not change after ACL reconstruction.^22^ In contrast, the current systematic review had a different focus, specifically looking at the association of biomarkers with outcome variables. Building on the data from the systematic review by Harkey et al,^22^ biomarkers of chondral metabolism were associated with PROMs and radiological outcomes (Table 3). Although encouraging, the available literature is nonetheless limited by heterogeneous outcome measures and small sample sizes.

It is worth noting that many of the included studies found associations between specific biomarker profiles^3,40^ or ratios^53,56,63^ and an outcome of interest. This may indicate that it is not one specific biomarker that is associated with the outcome but rather a combination of biomarkers. Interpreting multiple biomarkers together may allow assessment of both chondral anabolism and catabolism, or pro- and anti-inflammatory agents, for example, and may be more reflective of the net status or homeostasis at the cellular level within the knee. An “inflammatory phenotype” was proposed by Avila et al^4^ as one example of this concept.

If biomarkers are to become adopted clinically in the context of ACL reconstruction, there are a number of requirements. The ideal biomarker would be easily accessible; urine and serum biomarkers would be preferable because they avoid the need for arthrocentesis. If a synovial fluid marker were required, it would ideally need sampling only at the time of surgery to avoid the need for multiple joint aspirations. Other desirable attributes would be for a biomarker to be cheap and easy to measure, with high accuracy and precision of measurement. This has not been demonstrated in the current literature. Ideally, the marker would have a strong association with an important outcome demonstrated in multiple prospective series in differing patient cohorts. The outcome measured would also need to be something that could help guide treatment to optimize outcomes or, at a minimum, help counsel patients if the marker correlated with an unmodifiable factor. This may include using the biomarker to help guide timing of surgery, graft choice, or extra-articular procedures or counsel a patient about the probability of posttraumatic osteoarthritis. As one example of how this might be used clinically, elevated preoperative TTT levels (shown to be associated with cyclops lesion development)^32^ may lead surgeons to mitigate risk by taking a smaller-diameter quadricep tendon graft for male patients (another risk factor for cyclops development in other series).^21^ Even if this was a not modifiable factor, it may be beneficial to help with decision-making regarding return to sport, lifestyle, and occupation. For example, if a biomarker profile could accurately predict early-onset arthritis, this may be taken into consideration by patients and steer them away from occupations with high demands on the knee. Furthermore, accurate identification of patient subgroups at risk of an outcome like arthritis may provide a target group for therapeutic interventions.

### Limitations

This systematic review is limited by the quality of the included studies and marked heterogeneity in the biomarkers evaluated, timing of biomarker collection, reporting of concomitant knee injuries, and outcomes assessed. There may be other biomarkers strongly associated with ACL injuries; however, the lack of correlation to an outcome variable excluded such papers from the scope of this review. These other markers may still warrant investigation, so the markers listed here cannot be considered a comprehensive list of potential ACL-related biomarkers. The conclusions of this review have been tempered in light of these limitations, but it still provides a comprehensive update on this rapidly evolving topic and a platform for future work that appears justified based on the many promising biomarkers identified.

## Conclusion

Biomarkers in the blood, urine, and synovial fluid can be measured after ACL injury and reconstruction and have been associated with clinically important outcome measures after surgery. The current results highlight several biomarkers that may warrant further research to understand if these biomarkers can provide meaningful information in the clinical environment.

## Appendix

**Appendix Table A1:** MEDLINE Search Strategy

Search No.	Search Terms	Search Notes	Results
S1	Anterior Cruciate Ligament/	MeSH	11,660
S2	“anterior cruciate ligament*”		24,973
S3	ACL OR ACLR		19,613
S4	1 OR 2 OR 3		29,234
S5	Blood/	MeSH	53,764
S6	Blood OR bloods		3,997,776
S7	Plasma/	MeSH	24,156
S8	Plasma		984,856
S9	Serum/	MeSH	11,184
S10	Serum		1,163,613
S11	Urine/	MeSH	37,942
S12	Urine		381,615
S13	Synovial fluid/	MeSH	13,528
S14	“synovial fluid*”		18,724
S15	Biomarkers/	MeSH	324,330
S16	Biomarker*		693,576
S17	CTX-2		183
S18	Matrix metalloproteinases/	MeSH	10,592
S19	“matrix metalloproteinases” OR MMp		68,354
S20	TNF-related apoptosis-Inducing Ligand/	MeSH	5929
S21	TNF		199,434
S22	Interleukins/	MeSH	18,120
S23	Interleukin*		386,929
S24	Cartilage Oligomeric Matrix Protein/	MeSH	793
S25	“cartilage oligomeric matrix protein” OR COMP		4648
S26	Transforming Growth Factor beta/ OR Transforming growth factor beta1/ OR Latent TGF-beta Binding Proteins/	MeSH	64,016
S27	TGF		82,006
S28	Tissue Inhibitor OR Metalloproteinases/	MeSH	3600
S29	“tissue inhibitor or metalloproteinases” OR TIMP		14,688
S30	NTX		2279
S31	ARGS		3065
S32	Interferons/	MeSH	24,838
S33	Interferons OR IFN		152,113
S34	OR/5-33		
S35	4 AND 34		3670
S36	35 not (Animals/ not (Animals/ and Humans/))	MEDLINE human studies filter	2834
S37	Limit 36 to English language		2617

**Table A2:** List of Biomarker Abbreviations With a Summary of Biomarker Function

Abbreviation	Full Name	Basic Function	Included Study Reporting on Biomarker
Aggrecan	Aggrecan	Major proteoglycan in articular cartilage^28^	Pietrosimone^54^
ALT	Alanine transaminase (also known as alanine aminotransferase)	Enzyme found mostly in hepatocytes that catalyzes the reversible interconversion of l-alanine and 2-oxoglutalate to pyruvate and l-glutamate^41^; has also been found to be elevated in skeletal muscle diseases^49^	Kodama^32^
AST	Aspartate transaminase (also known as aspartate aminotransferase)	Enzyme found mostly in hepatocytes; catalyzes the transamination reaction between l-aspartate and 2-oxoglutarate into oxaloacetate and l-glutamate^31^; also found to be elevated in skeletal muscle diseases^49^	Kodama^32^
Bilirubin + biliverdin	Bilirubin and biliverdin	By-product of heme catabolism and is subsequently reduced to bilirubin^47^	Amano^3^
BMP-2	Bone morphogenic protein 2	Role in signaling osteoblast proliferation and differentiation^6^	Zysk^69^
C1,2C	Collagen type 1 and 2 cleavage product	By-product of type 1 and 2 collagen cleavage by collagenases^61^	Tourville^63^
C2C	Collagen type 2 cleavage product	By-product of cleavage of type 2 collagen cleavage by collagenases^61^	Lisee,^40^ Pietrosimone,^53,54^ Sobue,^56^ Tourville^63^
C2C/CPII	Collagen type 2 cleavage product/procollagen 2 carboxypeptide	Ratio of type 2 collagen degradation relative to synthesis^40^	Lisee,^40^ Pietrosimone^53^
C2C/KS	Collagen type 2 cleavage product/keratan sulfate	Ratio of C2C/KS in synovial fluid that may offer greater ability to identify early, pre-radiographic high-grade cartilage damage better than either biomarker individually^56,68^	Sobue^56^
C4S	Chondroitin-4-sulfate	In osteoarthritic cartilage, C6S is replaced by C4S^48^	Sobue^56^
C6S	Chondroitin-6-sulfate	Major glycosaminoglycan found in articular cartilage^48^	Sobue^56^
C6S/C4S	Chondroitin-6-sulfate/chondroitin-4-sulfate	Ratio of C6S to C4S, considered to be a marker of osteoarthritis progression^48^	Sobue^56^
COMP	Cartilage oligomeric matrix protein	Extracellular matrix protein that binds to aggrecan; considered to be a biomarker of matrix degeneration^40^	Latterman,^36^ Lisee^40^
CPII	Procollagen 2 C-propeptide	Marker of type 2 collagen synthesis^61^	Amano,^3^ Tourville^63^
CRP	C-reactive protein	Acute inflammatory protein synthesized primarily in the liver^57^	Kodama^32^
CTX-II	C-terminal cross-linked telopeptide of type 2 collagen	By-product of type 2 collagen and articular cartilage degradation^7^	Chmielewski,^7^ Latterman,^36^ Tourville^63^
FGF-2 (bFGF)	Fibroblast growth factor 2 (also known as basic fibroblast growth factor)	Growth factor promoting chondrogenesis, angiogenesis, wound healing, and granulation tissue formation; excess may contribute to onset or progression of osteoarthritis^4,24^	Avila,^4^ Markus^44^
IFN-γ	Interferon gamma	Pro-inflammatory cytokine that has been used as a marker of cartilage degeneration^3,62^	Amano,^3^ Inoue^25^
IL-1	Interleukin 1	Refers to IL-1a and IL-1b, 2 cytokines that play a major role in inflammation^26^	Gupta^19^
IL-1a	Interleukin 1 alpha	Pro-inflammatory cytokine produced by macrophages and epithelial cells^3^	Amano,^3^ Latterman^36^
IL-1b	Interleukin 1 beta	Pro-inflammatory cytokine produced by macrophages and epithelial cells^3^	Amano,^3^ Inoue,^25^ Latterman,^36^ Sullivan,^60^ Zysk^69^
IL-1Ra	Interleukin 1 receptor antagonist	Anti-inflammatory cytokine that inhibits the effect of IL-1b^4,26^	Amano,^3^ Avila,^4^ Latterman,^36^ Markus^44^
IL-2	Interleukin 2	Cytokine produced by activated T cells with a role in mediating the immune system by promoting growth and differentiation of T cells, T-helper cells, and NK cells^23^	Amano,^3^ Inoue^25^
IL-4	Interleukin 4	Cytokine produced by Th2 cells and mast cells; involved in B-cell activation, IgE switch, and suppression of Th1 cells^26^	Amano^3^
IL-6	Interleukin 6	Cytokine secreted by T cells, macrophages, and endothelial cells during infection or trauma, stimulating IL-10 and IL-1Ra to act as a negative feedback mechanism to inflammation^4,17^	Amano,^3^ Avila,^4^ Evans-Pickett,^15^ Gupta,^19^ Inoue,^25^ Markus,^44^ Pietrosimone,^53^ Sobue,^56^ Sullivan,^60^ Zysk^69^
IL-8	Interleukin 8	Cytokine produced by monocytes that attracts neutrophils, basophils, and T cells^23^	Amano,^3^ Inoue^25^
IL-10	Interleukin 10	Anti-inflammatory cytokine produced by Th2 cells and macrophages that inhibits Th1 cells, increases MHC expression on B cells, and stimulates mast cell growth^23^	Amano,^3^ Inoue^25^
IL-12p70	Interleukin 12p70	Active form of cytokine IL-12 produced by B cells and macrophages that induces NK-cell activation and Th1-cell differentiation^23^	Amano^3^
IL-13	Interleukin 13	Anti-inflammatory cytokine produced by T cells; acts to promote B-cell growth/differentiation, inhibit macrophage cytokine production, and inhibit Th1 cells^23^	Amano^3^
KS	Keratan sulfate	A glycosaminoglycan that is one of the constituent proteins of aggrecan when attached to a core protein^56^; levels are decreased in cartilage degradation^68^	Sobue^56^
MCP-1 (CCL2)	Monocyte chemotactic protein 1 (also known as chemokine ligand 2)	Chemokine that recruits monocytes, memory T cells, and dendritic cells to inflammatory sites^4,12^	Avila,^4^ Lisee,^40^ Markus^44^
MIP-1B	Macrophage inflammatory protein 1B	Chemokine produced by neutrophils that activates granulocytes; recruits neutrophiles/monocytes/macrophages/immature dendritic cells/Th1 cells to inflammatory sites^4^	Avila,^4^ Markus^44^
MMP-1	Matrix metalloproteinase 1	Metalloproteinase that degrades type 1, 2, 3, 7, 8, and 10 collagen and gelatin, aggrecan, and proteoglycan link proteins^12^	Amano,^3^ Latterman^36^
MMP-3	Matrix metalloproteinase 3	Metalloproteinase that degrades type 2, 4, 9, 10, and 11 collagen and proteoglycans, fibronectin, laminin, and elastin; can activate other MMPs^4,14^	Amano,^3^ Avila,^4^ Evans-Pickett,^15^ Latterman,^36^ Lisee,^40^ Markus,^44^ Pietrosimone^53^
MMP-9	Matrix metalloproteinase 9	Metalloproteinase that degrades type 4, 5, 7, 10, and 14 collagen and gelatin, aggrecan, elastin, fibronectin, laminin, and proteoglycan link protein^11^	Amano,^3^ Latterman^36^
NO	Nitric oxide	A free radical shown to have a role on potentiating cytokine and inflammation-induced bone resorption^65,69^	Zysk^69^
NTX-1	N-terminal telopeptide of type 1 collagen	Marker of breakdown of type 1 collagen and indicator of bone turnover^3,36^	Latterman,^36^ Amano^3^
RANTES (CCL5)	Regulated on activation, normal T cell expressed and presumably secreted (also known as chemokine ligand 5)	Chemokine that recruits leukocytes to the site of inflammation and activates natural killer cells^4,55^	Avila,^4^ Markus^44^
sGAG	Sulfated glycosaminoglycan	A class of long, linear polysaccharides that includes the chondroitin sulfate (eg, C4S, C6S) and keratan sulfate (KS) groups^64^	Amano,^3^ Latterman^36^
TIMP-1	Tissue inhibitor of metalloproteinases 1	Protein that inhibits MMPs; promotes cell growth of chondrocytes and has chondroprotective effect^4,18^	Avila,^4^ Markus^44^
TIMP-2	Tissue inhibitor of metalloproteinases 2	Protein that inhibits MMPs, favoring MMP-2^46^	Markus^44^
TNF-α	Tumor necrosis factor alpha	Pro-inflammatory cytokine produced by macrophages^1,3^	Amano,^3^ Gupta,^19^ Inoue,^25^ Zysk^69^
TSG-6	Tumor necrosis factor–stimulated gene 6 protein	Anti-inflammatory protein significantly upregulated in osteoarthritis progression^8^	Latterman^36^
TTT	Thymol turbidity test	A marker of inflammatory conditions such as chronic hepatitis, chronic infection, or collagen disease^32^	Kodama^32^
VEGF	Vascular endothelial growth factor	Growth factor that induces angiogenesis^4,45^	Avila,^4^ Markus^44^

## Notes

¶ References 3, 19, 25, 36, 40, 44, 53, 56, 60, 63, 69.

# References 3, 7, 15, 19, 36, 40, 53, 60, 63, 69.

** References 4, 7, 15, 19, 32, 36, 40, 44, 53, 56, 63, 69.

†† References 3, 4, 15, 19, 25, 36, 44, 56, 60, 69.
